# Fabrication of Highly Sensitive Porous Polydimethylsiloxane Pressure Sensor Through Control of Rheological Properties

**DOI:** 10.3390/polym16213075

**Published:** 2024-10-31

**Authors:** Yunseok Jang, Seung-Hyun Lee, Youn-Ki Lee, Inyoung Kim, Taik-Min Lee, Sin Kwon, Boseok Kang

**Affiliations:** 1 Department of Advanced Battery Manufacturing Systems, Korea Institute of Machinery & Materials, Daejeon 34103, Republic of Korea; 2 SKKU Advanced Institute of Nano Technology (SAINT), Department of Nano Science and Technology, and Department of Nano Engineering, Sungkyunkwan University (SKKU), Suwon 16419, Republic of Korea

**Keywords:** capacitive sensors, porous PDMS, bubbly PDMS, PDMS foam

## Abstract

In order to enhance the sensitivity of elastomers, pores were integrated into their structure. These pores facilitate the adjustment of thickness in response to external pressure variations, thereby improving the sensitivity of pressure sensors. Pores were introduced by emulsifying immiscible polydimethylsiloxane (PDMS) and water with a surfactant. By controlling the water content in the PDMS and water emulsion, we controlled the size, density, uniformity, and spatial distribution (2D or 3D) of the pores within the PDMS matrix. The presence of these pores significantly improved the sensitivity of PDMS under low external pressure conditions compared to high pressures. Specifically, porous PDMS exhibited approximately 10-times greater sensitivity under low-pressure conditions than non-porous PDMS. The effectiveness of porous PDMS was demonstrated through dynamic loading and unloading detection of a small Lego toy and monitoring of human heartbeats. These results highlight the efficacy of our pressure sensor based on porous PDMS, which is fabricated through a simple and cost-effective process using a PDMS and water emulsion. This approach is highly suitable for developing the ability to detect applied pressures or contact forces.

## 1. Introduction

Population aging is a notable global social phenomenon that is progressing rapidly. Currently, Japan, Finland, and Italy have the highest percentages of elderly people. Within the Organization for Economic Co-operation and Development (OECD), Greece, Korea, Poland, Portugal, Slovenia, and Spain are noted for their accelerated aging rates. Outside the OECD, Brazil, China, and Saudi Arabia are among the countries experiencing the fastest aging rates [1,2]. Caring for this aging population has become a global priority, with a strong emphasis on cost-effectiveness. Pressure sensors are particularly significant as they offer crucial, indirect information about major health conditions at minimal expense, monitoring pressures within the brain, blood, vessels, and organs [3,4]. Consequently, pressure sensors are anticipated to play a pivotal role in the care and management of the elderly population.

A pressure sensor is an electronic device that converts pressure signals into corresponding electrical signals, such as resistive, piezoelectric, triboelectric, or capacitive electrical signals [5,6]. Capacitive pressure sensors are particularly noted for their linear response, distinguishing then from resistive, piezoelectric, and triboelectric sensors, which often exhibit nonlinearity [7,8]. This distinction can be attributed to the relationship between capacitance per unit area and thickness, expressed by the following equation:

(1) $$C=\frac{\varepsilon_{r}\;\varepsilon_{o}\;S}{d}$$

where $\varepsilon_{r}$ is the relative dielectric constant of the dielectric layer, $\varepsilon_{o}$ is the permittivity of free space, *S* is the area of the electrode, and *d* is the thickness of the dielectric layer [9]. Capacitance per unit area varies inversely with thickness, and capacitive pressure sensors demonstrate excellent linear response to external stimuli.

Due to its significant thickness variation in response to external stimuli, elastomers such as polydimethylsiloxane (PDMS), the most commonly used types, were selected as the sensing material in this study. Achieving a sensitive sensor with a high signal-to-noise ratio requires the elastomer to change thickness readily even under weak forces. Previous research has explored enhancing sensitivity to external stimuli by introducing pores on the surface and within the structure of the elastomer [10,11,12,13]. Surface pores enhance the sensitivity of pressure sensors by creating microscopic voids where the elastomer can deform under external pressure. This effect has been demonstrated using pyramidal and wrinkled microstructures by Z. Bao et al. [10,11] and H.S. Lee et al. [12], respectively. Additionally, research has shown that internal pores within the elastomer also facilitate deformation in response to external forces [13,14,15]. These studies collectively indicate that introducing pores either within or on the surface of an elastomer enhances its responsiveness to external stimuli. As external force increases, surface pores on the elastomer deform, enlarging the contact area between the electrode and the elastomer. In contrast, when pores are situated inside the elastomer, the change in contact area between the electrode and the elastomer diminishes with increasing external force compared to surface pores. Consequently, when pores are internal to the elastomer, a more linear response is observed upon application of external force compared to when pores are located on the elastomer surface.

In this study, we propose a method to enhance sensitivity to applied force by creating pores within the elastomer. By carefully controlling the rheological properties of the elastomer, we successfully fabricated elastomers with two- or three-dimensional internal pores. We found pore formation transitions from two-dimensional (2D) to three-dimensional (3D) under specific conditions. To evaluate the impact of internal pores on the pressure sensitivity of the elastomer, a comparative analysis was performed using an elastomer without internal pores.

## 2. Experiment

### 2.1. Preparation of PDMS with Pores

Polydimethylsiloxane (PDMS, Sylgard 184 silicon elastomer kit, Dow Corp., Midland, MI, USA) and water, which are normally immiscible, were emulsified using a surfactant (BYK-331, silicon surface additive, BYK Corp., Wesel, Germany) to create an emulsion similar to milk. This emulsion was utilized to fabricate PDMS with pores. We added 10 wt% BYK-331 to water and adjusted the ratio of PDMS to water to determine the composition that yielded the best pressure response. The weight ratios of PDMS–hardener–water tested were 10:1:0, 10:1:0.5, 10:1:1, 10:1:2, 10:1:3, and 10:1:4, respectively. Emulsions of various ratios were prepared using a planetary mixer (Thinky Corp., Laguna Hills, CA, USA) and 10 mm zirconia balls. PDMS and water emulsions at various ratios were poured into a stainless-steel mold with dimensions of depth 60 mm, width 60 mm, and height 1 mm. An imide film was placed over the mold to slow down water evaporation during the curing process. Excess material was removed using a scraper, and the emulsion was cured on a hot plate at 373.15 K for 60 min. After curing, residual water in the PDMS sheets was eliminated by placing them in a vacuum oven at 393.15 K for 60 min. The thickness of the PDMS sheets produced from emulsions with ratios of 10:1:0, 10:1:0.5, 10:1:1, 10:1:2, 10:1:3, and 10:1:4 were measured as follows: 1.1 ± 0.1, 1.2 ± 0.1, 1.1 ± 0.1, 1.1 ± 0.2, 1.1 ± 0.1, and 1.2 ± 0.1 mm, respectively (See Supplementary Materials).

### 2.2. Characteristics of Pressure Sensors Made from Various PDMS

The pressure sensors were fabricated by cutting various PDMS sheets using a 19 mm diameter circular punch. These sheets were then positioned between a circular SUS electrode measuring 1.14 mm in diameter and 1 mm in height (top electrode) and a square brass electrode measuring 30 mm × 30 mm × 15 mm (length × width × height, bottom electrode). The rigid SUS electrode was used as the top electrode to prevent electrode deformation during measurements. The capacitive response to different stimuli of the PDMS sheets was measured using an Agilent 4980A Precision LCR meter. The pressure sensing capabilities of the sensors were evaluated using a computer-controlled homemade sensor measurement system equipped with a z-axis moving stage (LNR50SE, Thorlabs Corp., Newton, NJ, USA) with a 50 mm movement range and a force gauge (M7-05, Mark-10 Corp., Copiague, NY, USA) with a capacity of 250 gF. The moving stage operated at a speed of 1 mm/s. The load registered by the force gauge was measured, and the corresponding pressure was calculated by dividing the applied force by the area of the top electrode.

## 3. Results and Discussion

**Figure 1:**
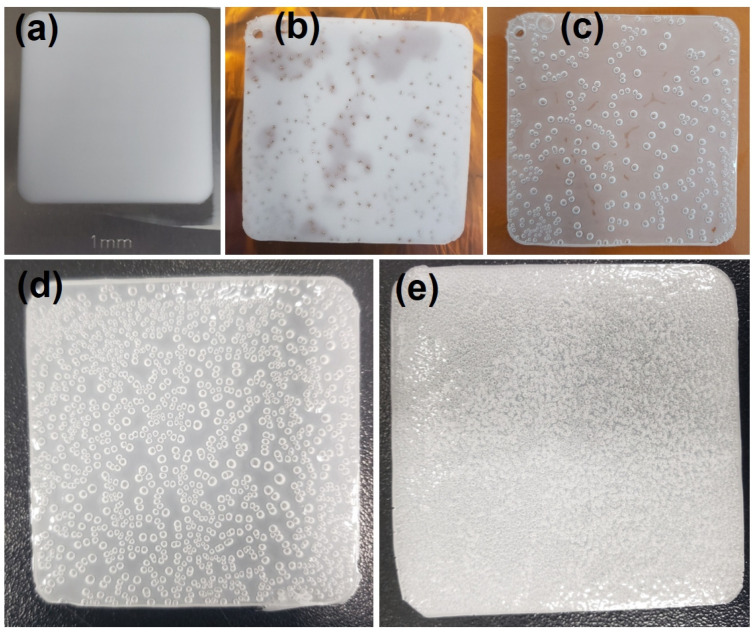
Digital camera images of (**a**) the SUS mold filled with PDMS and water emulsion at a ratio of 10:1:1; (**b**) the PDMS sheet after crosslinking at a ratio of 10:1:1; (**c**) the PDMS sheet after drying at a ratio of 10:1:1; (**d**) PDMS sheet made at a ratio of 10:1:2; (**e**) PDMS sheet made at a ratio of 10:1:3 after baking in a vacuum oven. The size of each PDMS sheet was approximately 60 mm × 60 mm.

**Figure 2:**
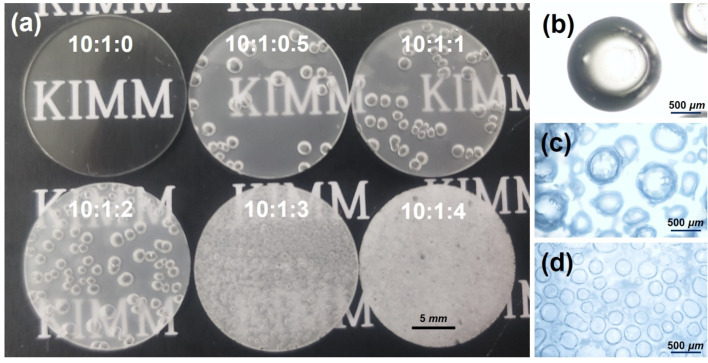
(**a**) A digital camera image of PDMS sheets made of 10:1:0, 10:1:0.5, 10:1:1, 10:1:2, 10:1:3, and 10:1:4, optical microscopy images of PDMS sheets made at (**b**) 10:1:2, (**c**) 10:1:3, and (**d**) 10:1:4 ratios.

Figure 1a–c shows the process of fabricating a PDMS sheet with pores. As shown in Figure 1b,c, the PDMS sheet formed from PDMS and water emulsion retained some residual water even after cross-linking. Therefore, residual water on the PDMS sheet was removed using a vacuum oven. Figure 2a presents a digital camera image of various PDMS sheets cut using a 19 mm diameter circular punch. This image shows that the turbidity of PDMS increases with higher water content. This increase in turbidity is attributed to an elevated concentration of BYK-331 surfactant. The pore sizes of the PDMS sheets prepared with ratios 10:1:0.5, 10:1:1, and 10:1:2 were similar, while pore density increased with higher water content. Figure 2b shows an optical microscopy image (Eclipse, Nikon corp., Tokyo, Japan) of the PDMS sheet prepared at a 10:1:2 ratio, with a pore size of 1633 ± 201 µm. Because the pore sizes are similar between PDMS sheets prepared at 10:1:0.5, 10:1:1, and 10:1:2 ratios, only an image of the 10:1:2 is shown. The pore size of the 10:1:3 ratio PDMS sheet was uniformly and densely packed. The pore size of the 10:1:4 ratio PDMS sheet was smaller and denser compared to the 10:1:3 ratio PDMS sheet. The pore sizes of 10:1:3 and 10:1:4 were 459 ± 126 and 247 ± 48 µm, respectively. The pores in the 10:1:0.5, 10:1:1, and 10:1:2 exhibit a 2D pore distribution, characterized by larger pore sizes compared to the 10:1:3, which exhibits a 3D pore distribution. The 10:1:4 pores were also expected to have a 3D distribution but were not clearly visible under an optical microscopy due to the turbidity of the PDMS sheet. Density measurements were performed to evaluate the porosity levels of various PDMS sheets. The densities of 10:1:0, 10:1:0.5, 10:1:1, 10:1:2, 10:1:3, and 10:1:4 were 1059 ± 5, 882 ± 7, 864 ± 1, 828 ± 4, 744 ± 8, and 570 ± 5 kg/m^3^, respectively. As the added water content increased, the density of the PDMS sheets decreased, indicating increased porosity. The PDMS sheet with a 10:1:4 ratio exhibited the lowest density, suggesting that there were pores not only on the surface but also inside.

**Figure 3:**
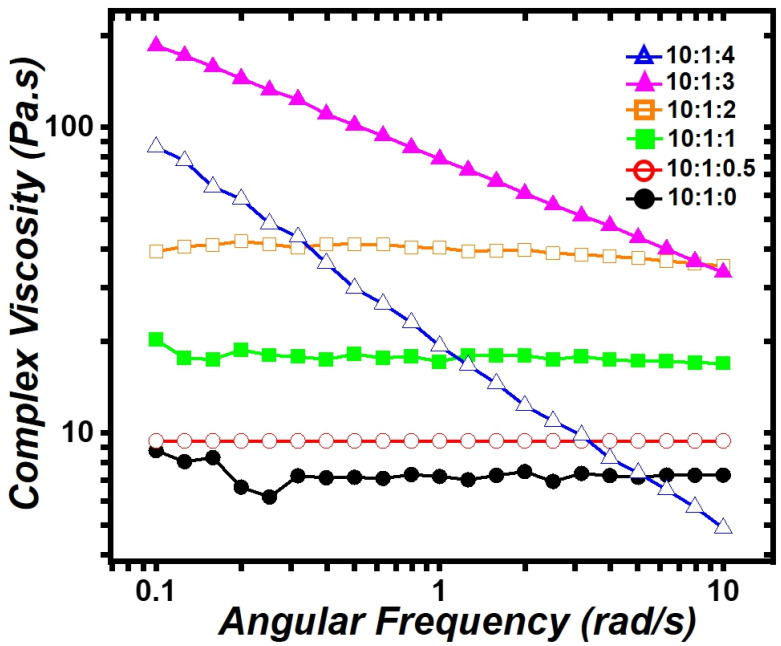
The complex viscosities of PDMS and water emulsions at various ratios are shown as follows: 10:1:0 (filled circles, black), 10:1:0.5 (open circles, red), 10:1:1 (filled rectangles, green), 10:1:2 (open rectangles, orange), 10:1:3 (filled triangles, magenta), and 10:1:4 (open triangles, blue). Inset: A digital camera image of a sensor consisting of a sandwich structure of electrode/sensor material/electrode.

Pores are voids that remain when water evaporates from water droplets within a PDMS and water emulsion. Since water droplets inside PDMS can influence the viscosity of PDMS and water emulsions, viscosity measurements were conducted using an Ares-G2 rheometer (25 mm parallel plate, TA Instruments, New Castle, DE, USA). Figure 3 shows the complex viscosity of various PDMS and water emulsions. The complex viscosity of 10:1:0, 10:1:0.5, 10:1:1, and 10:1:2 increases with higher water content. This increase in viscosity is attributed to the greater resistance caused by increased granular stress within the PDMS and water emulsion due to higher water content [16]. This phenomenon occurs because the water in PDMS and water emulsion exists as small particles within the PDMS matrix due to the surfactants, similar to water in oil [17,18]. This resistance prevents water from escaping the emulsion, increasing the pore density of PDMS sheets at 10:1:0.5, 10:1:1, and 10:1:2 ratios. The complex viscosity of PDMS sheets at 10:1:3 and 10:1:4 ratios exhibits distinct behavior compared to the other ratios. The complex viscosity at 10:1:0.1, 10:1:0.5, 10:1:1, 10:1:2 ratios exhibits Newtonian-fluid behavior, whereas, at 10:1:3 and 10:1:4 ratios, it shows non-Newtonian-fluid behavior. This transition from Newtonian-fluid to non-Newtonian-fluid behavior is a well-known phenomenon observed in emulsions of water and oil. The transformation of PDMS and water emulsions is expected to follow this principle. It has been widely established theoretically and experimentally that oil and water, which are Newtonian fluids, transform into non-Newtonian fluids when creating an oil and water emulsion [16,17,18,19]. Therefore, no further detailed explanation is necessary here. The shear-thinning phenomena observed at 10:1:3 and 10:1:4 ratios is generally due to a decrease in average cluster size with increasing shear rate. Deformation and compaction of clusters within the emulsion may contribute to shear-thinning behavior [18]. The PDMS and water emulsions depicted in Figure 2 and Figure 3 show that the shape of the PDMS sheet and the viscoelastic properties of the emulsion change as the water content increases above a certain threshold. To distinguish these differences above the threshold, the PDMS sheets were classified into “bubbly PDMS” and “PDMS foam”. “Bubbly PDMS” is produced at ratios less than 10:1:2, while “PDMS foam” is produced at ratios greater than 10:1:3. The complex viscosity of 10:1:4 exhibits different behavior compared to other emulsions in Figure 3. At high angular frequencies, the complex viscosity of 10:1:4 becomes very low. This is most likely because excess water droplets easily disrupt the PDMS structure when the rheometer disk oscillates at high angular frequencies [19]. The decrease in complex viscosity in 10:1:4 compared to 10:1:3 is also due to the addition of too much water.

**Figure 4:**
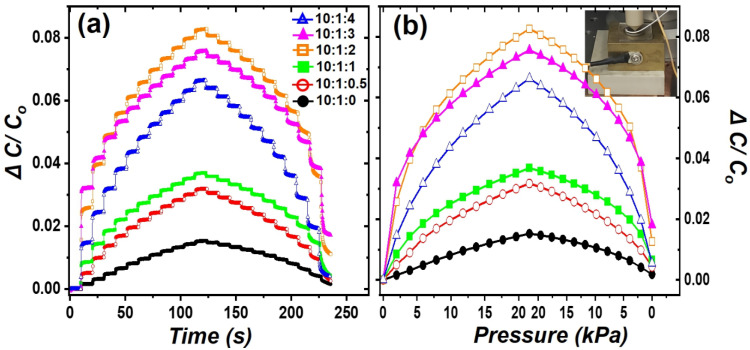
Relative capacitance change (Δ*C/C*_0_) as a function of (**a**) the step-by-step force in the 0~21.56 kPa range and (**b**) the normal force for sheets of 10:1:0 (filled circles, black), 10:1:0.5 (open circles, red), 10:1:1 (filled rectangles, green), 10:1:2 (open rectangles, orange), 10:1:3 (filled triangles, magenta), and 10:1:4 (open triangles, blue) ratios.

To characterize the piezoelectric response of PDMS sensing materials, the step pressure response performance of various PDMS sheets was evaluated for forces ranging from 0 to 21.56 kPa. As shown in Figure 4a, the relative capacitive change (Δ*C/C*_0_) was measured while applying a step force to the PDMS sensing materials between the top and bottom electrode for 10 s. It was observed that Δ*C/C*_0_ increased as the step force increased, as described by Equation (1). PDMS sheets of 10:1:2 and 10:1:3 show the best force response in Figure 4. Among them, the PDMS sheet with the 10:1:2 ratio exhibits the best performance among bubbly PDMS, while the PDMS sheet with the 10:1:3 ratio shows the best performance among PDMS foams. Although the force response properties of the PDMS sheet at 10:1:2 are better than those at 10:1:3, there are variations in bubble density and size distribution depending on location, as shown in Figure 1d,e. These differences lead to varying force response characteristics depending on location. Consequently, the PDMS sheet at the 10:1:3 ratio exhibits a uniform distribution of bubbles throughout and consistent force response characteristics. Therefore, the ratio of 10:1:3 was chosen as the optimal configuration for subsequent experiments.

**Figure 5:**
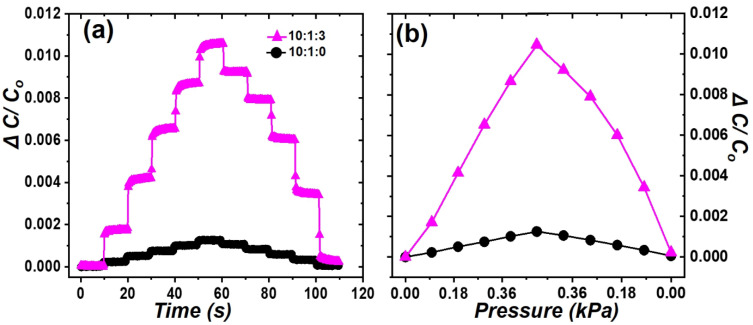
Relative capacitance change (Δ*C/C*_0_) as a function of (**a**) the step-by-step force in the 0~0.49 kPa range and (**b**) the normal force for sheets of 10:1:0 (filled circles, black) and 10:1:3 (filled triangles, magenta) ratios.

It can be seen that the early and late Δ*C/C*_0_ slopes of PDMS sheets at 10:1:2, 10:1:3, and 10:1:4 are different in Figure 4b. Specifically, the Δ*C/C*_0_ value for 10:1:3 is 0.057 at 9.8 kPa and 0.075 at 21.56 kPa. This indicates that 76% of the total capacitance change occurs at 45% of the total applied force, which is highly advantageous for detecting low pressures. This is because the pores inside PDMS can be easily compressed at low initial pressures, while the degree of compression decreases above a certain pressure threshold. The force response of PDMS sheets with 10:1:0 and 10:1:3 ratios was evaluated for low forces (i.e., from 0 to 0.49 kPa) in Figure 5. This demonstrates that the PDMS sheet with 10:1:3 ratio exhibits excellent response properties even at low forces.

The pressure sensitivity, defined as the *G-factor*, of the PDMS pressure sensors was determined by the slope of Δ*C/C*_0_ versus the normal pressure [14,20]. As shown in Figure 4b, the PDMS sheet with the 10:1:3 ratio initially exhibits high pressure sensitivity, but the sensitivity decrease as the force increases. In Figure 5b, the *G-factors* for 10:1:0 and 10:1:3 at low pressure (i.e., below 0.5 kPa) were 0.00259 and 0.022 kPa^−1^, respectively. In Figure 4b, the G-factors for 10:1:0 and 10:1:3 at high pressure (i.e., more than 10 kPa) were 0.000574 and 0.00146 kPa^−1^, respectively. Porous PDMS sheet showed an approximately 10-times improvement in low-pressure sensitivity compared to the non-porous PDMS sheet. These results clearly indicate that porous PDMS is very useful for low-pressure sensing applications.

**Figure 6:**
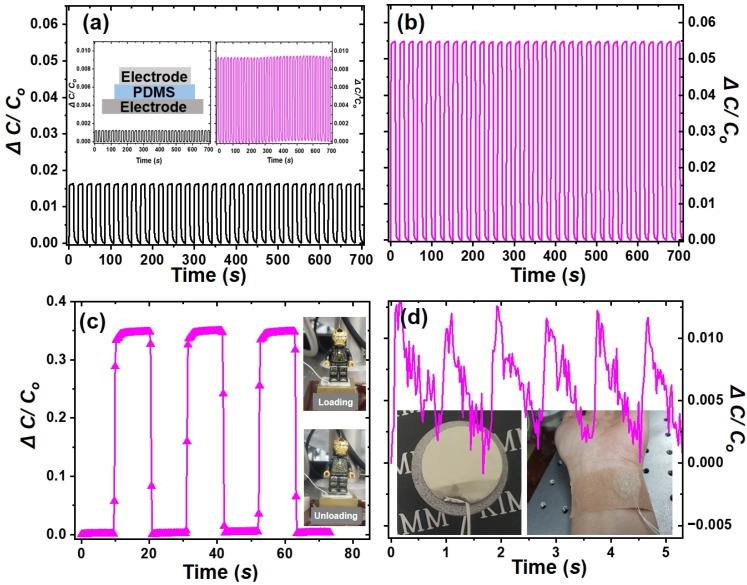
Durability test under an applied pressure of 11.76 kPa over more than 35 cycles for sheets with (**a**) 10:1:0 and (**b**) 10:1:3 ratios. Capacitance response plot of a 10:1:3 sheet (**c**) to dynamic loading and unloading cycles of a small Lego toy and (**d**) Capacitance response plot of a 10:1:3 sheet to detecting human heartbeats. Inset: Durability test under an applied pressure of 0.49 kPa over more than 35 cycles for sheets with 10:1:0 (left) and 10:1:3 (right) ratios in (**a**). And a schematic diagram of the sensor is inset. The inset in (**d**) shows a digital image of the sensor, which has a metal/PDMS/metal sandwich structure and is attached to the wrist with 3M medical tape.

The operational reliability of the pressure sensor fabricated from porous PDMS is shown in Figure 6. Device durability was evaluated by measuring Δ*C/C*_0_ over 33 repeated dynamic load and unload cycles at an applied pressure of 11.76 kPa, as shown in Figure 6a,b. Pressure sensors with compositions of 10:1:0 and 10:1:3 exhibited Δ*C/C*_0_ values of 0.016 and 0.054, respectively. The inset in Figure 6a shows the dynamic load and unload cycles of these sensors under an applied pressure of 0.49 kPa. Pressure sensors with compositions of 10:1:0 and 10:1:3 exhibited Δ*C/C*_0_ values of 0.0012 and 0.0094, respectively. Compared to non-porous PDMS, porous PDMS exhibited the Δ*C/C*_0_ values that were 3.4 times higher at 11.76 kPa and 7.8 times higher at 0.49 kPa, indicating improved sensitivity to detect signals at low pressures.

To verify the effectiveness of porous PDMS, Figure 6c shows the dynamic load and unload cycles of a small LEGO toy (mass: 3.9 g), demonstrating a high signal-to-nose ratio in pressure measurements. This demonstrates that pressure sensors fabricated from porous PDMS exhibit high sensitivity. Figure 6d shows heartbeat signal detection, demonstrating their capability to detect small signals such as heartbeats. These results indicate that our porous PDMS pressure sensor is suitable for detecting applied pressures or contact forces.

## 4. Conclusions

In this study, we propose a method to introduce pores into PDMS to enhance the sensitivity of pressure sensors. By increasing the amount of water in PDMS and water emulsion, we observed a transition from Newtonian to non-Newtonian fluid beyond a certain content threshold. Below this threshold, “bubbly PDMS” is produced, while, above it, “PDMS foam” is generated. Larger pores significantly enhance sensitivity. Bubbly PDMS, characterized by its large pores, demonstrates heightened sensitivity, making it an excellent option for pressure sensors. However, it encounters challenges such as poor uniformity and variability in electrical properties across different spatial locations. Conversely, PDMS foam has smaller pores and shows lower sensitivity as a pressure sensor compared to bubbly PDMS with larger pores. However, it offers advantages such as excellent uniformity and consistent electrical properties across multiple locations.

The pores within PDMS play a crucial role in enhancing sensitivity, enabling more accurate detection signals at lower pressures. Porous PDMS sheets have been demonstrated to achieve approximately 10-times greater sensitivity at low pressures compared to non-porous PDMS. The effectiveness of PDMS pores, particularly in PDMS foam, was confirmed through the detection of dynamic loading and unloading of a small Lego toy, as well as the monitoring of human heartbeats. These results demonstrate that our porous pressure sensor, fabricated through a simple and cost-effective process using PDMS and water emulsion, serves as an effective device for detecting applied pressures or contact forces. Ultimately, we envision that porous PDMS can play a significant role in addressing the needs of the growing elderly population.

## Data Availability

The data that support the findings of this study are available from the corresponding author upon reasonable request.

## Supplementary Materials

The following supporting information can be downloaded at: https://www.mdpi.com/article/10.3390/polym16213075/s1: Figure S1. Digital camera images of PDMS sheet thickness measurements at 10:1:0, 10:1:0.5, 10:1:1, 10:1:2, 10:1:3, and 10:1:4 ratios.
