# Supplementary material for: Fabrication of Highly Sensitive Porous Polydimethylsiloxane Pressure Sensor Through Control of Rheological Properties
Source: Polymers (Basel). 2024 Oct 31;16(21):3075. doi: 10.3390/polym16213075 (PMC11548559; doi:10.3390/polym16213075)
Supplement: Supplementary file 1 [file polymers-16-03075-s001.zip › polymers-3287165-supplementary.pdf]

Fabrication of highly sensitive porous PDMS pressure sensor through control of rheological properties

Yunseok Jang[1,a)], Seung-Hyun Lee[1)], Youn-Ki Lee[1)], Inyoung Kim[1)], Taik-Min Lee[1)], Sin Kwon[1)], and Boseok Kang[2,b)]

[1]Department of Advanced Battery Manufacturing Systems,
Korea Institute of Machinery & Materials, Daejeon, 34103, Korea.

[2]SKKU Advanced Institute of Nano Technology (SAINT), Department of Nano Science and Technology, and Department of Nano Engineering, Sungkyunkwan University (SKKU), Suwon, 16419, Korea

Keywords: capacitive sensors; porous PDMS; bubbly PDMS; PDMS foam

* To whom all correspondence should be addressed.

E-mail: [a)]yjang@kimm.re.kr , [b)]bskang88@skku.edu

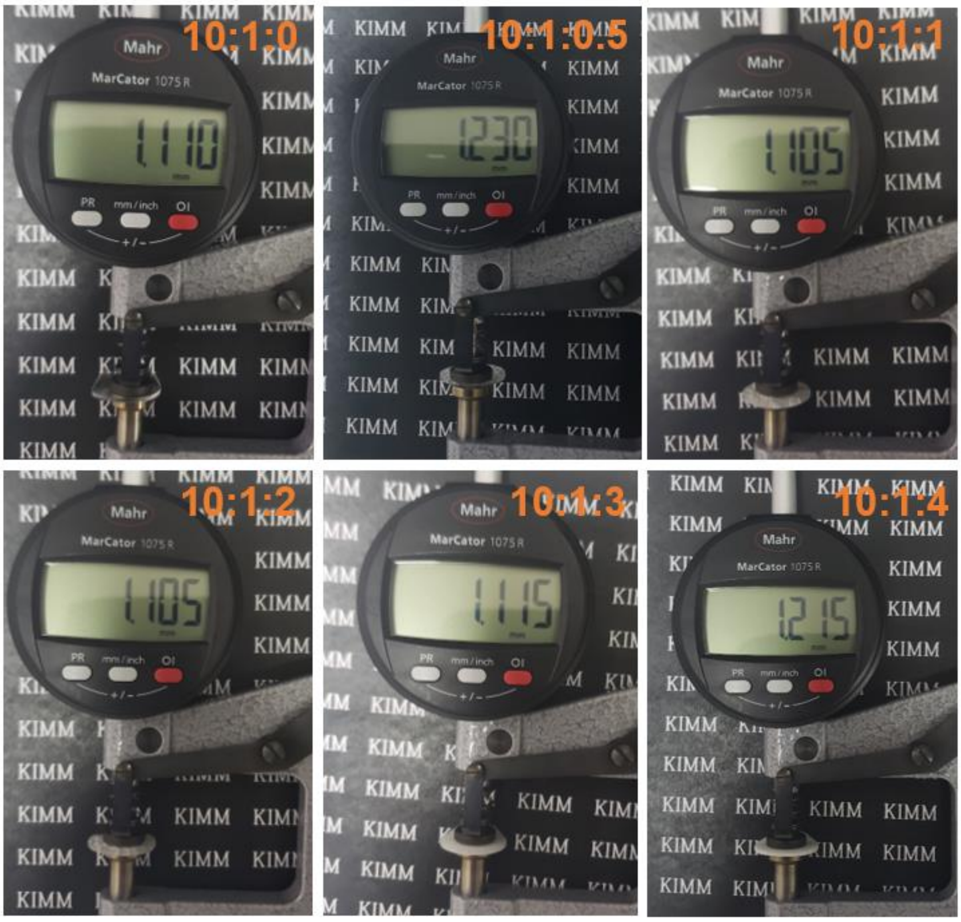

Figure S1. Digital camera images of PDMS sheet thickness measurements at 10:1:0, 10:1:0.5, 10:1:1, 10:1:2, 10:1:3, and 10:1:4 ratios.
